# Robust PMSM Speed Control for EV Traction Drives: A FOPSO-Optimized Hybrid Fuzzy Fractional-Order PI Strategy

**DOI:** 10.3390/s26051461

**Published:** 2026-02-26

**Authors:** Chih-Chung Chiu, Wei-Lung Mao, Feng-Chun Tai

**Affiliations:** Department of Electrical Engineering, Graduate School of Engineering Science and Technology, National Yunlin University of Science and Technology, 123 University Road, Section 3, Douliou 64002, Yunlin, Taiwan

**Keywords:** permanent magnet synchronous motor (PMSM), electric vehicles (EVs), fractional-order control, fuzzy logic, particle swarm optimization (PSO), CarSim co-simulation, sensor noise, robustness

## Abstract

High-performance speed control of Permanent Magnet Synchronous Motor (PMSM) drives in Electric Vehicle (EV) applications faces significant challenges due to inherent nonlinearities, parameter variations, and signal non-idealities such as sensor noise and measurement latency. To address these issues, this paper proposes a robust PI-based Fractional-Order PSO-Fuzzy Weight Controller (PI-FOPSOFWC). The proposed strategy integrates a fractional-order PI (FOPI) core to ensure iso-damping robustness, a fuzzy inference mechanism for online gain scheduling against nonlinear load dynamics, and a novel Fractional-Order Particle Swarm Optimization (FOPSO) algorithm for optimal parameter tuning. A key contribution of this study is the validation of the control strategy within a high-fidelity co-simulation framework coupling MATLAB/Simulink with CarSim 2023, which incorporates realistic vehicle dynamics and time-varying road loads unavailable in conventional simplified simulations. Co-simulation results demonstrate that the proposed controller effectively eliminates overshoot in step responses and maintains stability under significant parameter mismatches (2.0× inertia). Furthermore, under the EPA urban driving cycle, the proposed method reduces the speed tracking Root Mean Square Error (RMSE) by 75.0% compared to the standard PI controller. Computational complexity analysis further confirms the feasibility of the proposed algorithm for real-time implementation in commercial EV traction drives.

## 1. Introduction

The electrification of the automotive industry has accelerated rapidly in recent years, driven by global initiatives to reduce carbon emissions and reliance on fossil fuels. Among the various electric propulsion technologies, the PMSM has emerged as the preferred choice for EV traction drives due to its superior power density, high efficiency, and high torque-to-inertia ratio compared to induction or switched reluctance motors [1,2].

However, high-performance speed control of PMSM drives in EV applications faces significant challenges. The vehicle traction system is inherently nonlinear and subject to various uncertainties, including parameter variations (e.g., inductance saturation, resistance drift due to temperature), unmodeled dynamics, and complex external load disturbances [3,4]. Furthermore, in practical implementations, signal non-idealities such as sensor measurement noise and communication latency can severely degrade the stability and bandwidth of the closed-loop system, leading to torque ripples and reduced ride comfort.

The classical Proportional–Integral (PI) controller, typically implemented within the Field-Oriented Control (FOC) framework [5,6], remains the industry standard due to its simplicity. However, the fixed-gain PI controller lacks the adaptability to handle the highly nonlinear and time-varying dynamics of an EV powertrain. To address this, various advanced control strategies have been proposed, including sliding mode control, model predictive control, and intelligent control schemes such as neuro-fuzzy and fractional-order adaptive controllers [7]. Recently, an array of advanced robust control and evolutionary optimization techniques—such as variable fractional-order architectures, adaptive fuzzy logic enhancements, and improved particle swarm optimization (PSO) methodologies—have been actively explored to further elevate PMSM drive performance under severe parameter mismatches and complex EV load conditions [8,9,10,11,12,13,14,15,16,17]. Among these, Fractional-Order Control has gained significant attention. By generalizing the integral and differential orders from integers to real numbers, fractional-order controllers (e.g., $PI^{\lambda }D^{\mu }$) provide additional degrees of freedom for loop shaping. This grants the system the “iso-damping” property, ensuring that the phase margin remains constant despite gain variations, which provides superior robustness compared to integer-order counterparts [18,19,20].

Despite the theoretical advantages of fractional-order controllers, tuning their parameters ($K_{p},K_{i},\lambda$) is a complex non-convex optimization problem. Metaheuristic algorithms, such as Particle Swarm Optimization (PSO), are widely used for automated tuning [21,22]. However, standard PSO often suffers from premature convergence, particularly in highly nonlinear and non-convex control optimization problems [23]. Moreover, most existing studies validate their control strategies using simplified simulations with constant step loads, which fail to capture the realistic, nonlinear road load dynamics experienced by an actual vehicle.

To bridge these gaps, this paper proposes a robust PI-based Fractional-Order PSO-Fuzzy Weight Controller (PI-FOPSOFWC). This hybrid strategy integrates the robustness of fractional calculus, the adaptability of fuzzy logic, and the global search capability of an enhanced optimization algorithm. Furthermore, to ensure rigorous validation, the system is tested within a high-fidelity co-simulation framework coupling MATLAB/Simulink (R2023b) with CarSim 2023, a specialized vehicle dynamics engine [24].

The main contributions of this paper are summarized as follows:

1. A fractional-order PI (FOPI) control architecture is designed to provide the “iso-damping” property, ensuring consistent transient response despite parameter mismatches and sensor non-idealities.
2. A fuzzy inference mechanism is integrated to perform online gain scheduling, effectively compensating for nonlinear load variations derived from vehicle dynamics.
3. An improved Fractional-Order PSO (FOPSO) algorithm is implemented, utilizing a discrete-time fractional memory term (Grünwald-Letnikov approximation) to enhance population diversity and avoid premature convergence during parameter tuning.
4. A realistic co-simulation platform is established using CarSim 2023 and the EPA Urban Cycle. Simulation results demonstrate that the proposed controller reduces the Root Mean Square Error (RMSE) by **75.0%** compared to the standard PI benchmark under realistic driving conditions.

The remainder of this paper is organized as follows: Section 2 describes the system modeling and co-simulation architecture. Section 3 details the design of the PI-FOPSOFWC. Section 4 presents the optimization algorithms. Section 5 provides the simulation results and discussion, followed by conclusions in Section 6.

## 2. System Model and Co-Simulation Architecture

This section details the mathematical formulation of the PMSM servo drive and the structural architecture of the proposed closed-loop system, designed for high-performance EV traction applications. To ensure a realistic assessment of sensor performance and control robustness under dynamic road conditions, the system is implemented within a co-simulation framework that integrates the control architecture (MATLAB/Simulink) with high-fidelity vehicle dynamics software (CarSim 2023).

The drive system consists of four major subsystems: (1) the Controller (FOPI + Fuzzy + PSO), (2) the plant (Voltage Source Inverter and PMSM), (3) the Sensor Module providing speed feedback with modeled non-idealities, and (4) the external Vehicle Dynamics Model (CarSim) supplying dynamic road load torques ($T_{L}$). This setup adopts the classical Field-Oriented Control (FOC) principle, which remains a benchmark strategy for EV propulsion systems.

### 2.1. Drive System Components and Mathematical Model

#### 2.1.1. PI-Based Fractional-Order PSO-Fuzzy Weight Controller (PI-FOPSOFWC)

The PI-FOPSOFWC serves as the central speed controller. The core control structure is derived from the generalized fractional-order $PI^{\lambda }D^{\mu }$ controller proposed by Podlubny [18]. However, in electric vehicle traction drives, the derivative action ($D^{\mu }$) is susceptible to high-frequency sensor noise amplification, which can lead to torque ripples and instability. Therefore, this study adopts a Fractional-Order PI (FOPI) configuration by setting the derivative order μ=0.

The resulting control law $u_{c}\left(t\right)$ (corresponding to the reference torque current $i_{q}^{*}$) is formulated as:

(1) uc(t)=Kp(e,e˙)e(t)+Ki(e,e˙)Dt−λ0Ce(t),

where $K_{p}$ and $K_{i}$ are the time-varying proportional and integral gains adjusted by the fuzzy inference mechanism. The term Dt−λ0C denotes the fractional-order integral operator of order λ (0<λ<2). Based on the Caputo definition, the operator is defined as:

(2) Dt−λ0Cf(t)=1Γ(λ)∫0tf(τ)(t−τ)1−λdτ,

where Γ(·) is the Gamma function. The fractional order λ is treated as a key decision variable within the optimization search space, determined by the FOPSO algorithm (typically converging to λ≈1.02) to provide additional phase compensation that indirectly compensates for unmodeled nonlinear dynamics.

The controller processes the speed tracking error e(t), which serves as the input to the control law:

(3) $$e\left(t\right)=r\left(t\right)-\omega_{m}^{\mathrm{meas}}\left(t\right),$$

where r(t) represents the target reference speed command, and $\omega_{m}^{\mathrm{meas}}\left(t\right)$ is the measured mechanical rotor speed feedback provided by the sensor module.

#### 2.1.2. Voltage Source Inverter (VSI)

The VSI converts DC power from the traction battery into three-phase AC voltages using Space Vector Pulse Width Modulation (SVPWM). To simplify the control design while retaining the essential dynamics, the inverter is modeled as a unity-gain first-order lag system. This control-oriented approximation is widely adopted in EV drive control studies where switching harmonics are not the primary focus, allowing the analysis to concentrate on closed-loop dynamic behavior and robustness. This approximation accounts for the cumulative latency caused by the digital computation and the PWM sample-and-hold effect [25]:

(4) $$V_{dq}^{actual}\left(s\right)=\frac{1}{1+\tau_{s}s}V_{dq}^{ref}\left(s\right),$$

where $V_{dq}^{actual}\left(s\right)$ and $V_{dq}^{ref}\left(s\right)$ denote the actual and reference voltage vectors in the s-domain. The time constant is approximated as $\tau_{s}\approx 1.5T_{sw}\approx 150\;\mu s$ (assuming a switching frequency $f_{sw}=10\;kHz$), representing the effective delay rather than the minimum on-time.

#### 2.1.3. Permanent Magnet Synchronous Motor (PMSM)

The dynamic equations of the surface-mounted PMSM in the dq rotating reference frame are expressed as:

(5) $$\begin{array}{cc} v_{d} & =R_{s}i_{d}+L_{d}\frac{di_{d}}{dt}-\omega_{e}L_{q}i_{q},\\ v_{q} & =R_{s}i_{q}+L_{q}\frac{di_{q}}{dt}+\omega_{e}\left(L_{d}i_{d}+\psi_{f}\right), \end{array}$$

where $v_{d}$ and $v_{q}$ are the *d*- and *q*-axis stator voltages; $i_{d}$ and $i_{q}$ are the *d*- and *q*-axis stator currents; $R_{s}$ represents the stator resistance; $L_{d}$ and $L_{q}$ denote the *d*- and *q*-axis inductances; $\omega_{e}$ is the electrical angular velocity; and $\psi_{f}$ is the permanent magnet flux linkage.

The electromagnetic torque $T_{e}$ is given by:

(6) $$T_{e}=\frac{3}{2}p\left[\psi_{f}i_{q}+\left(L_{d}-L_{q}\right)i_{d}i_{q}\right],$$

where *p* denotes the number of pole pairs. The mechanical motion is governed by:

(7) $$J_{m}\frac{d\omega_{m}}{dt}+B\omega_{m}=T_{e}-T_{L},$$

where $J_{m}$ is the rotor inertia, *B* is the viscous friction coefficient, and $T_{L}$ is the mechanical load torque. By applying the Laplace transform to (7), the rotor speed dynamics in the s-domain can be expressed as:

(8) $$\Omega_{m}\left(s\right)=\frac{T_{e}\left(s\right)-T_{L}\left(s\right)}{J_{m}s+B},$$

where $\Omega_{m}\left(s\right)$, $T_{e}\left(s\right)$, and $T_{L}\left(s\right)$ are the Laplace transforms of the rotor speed, electromagnetic torque, and load torque, respectively. This transfer function highlights the low-pass filtering effect of the mechanical inertia on torque disturbances.

#### 2.1.4. Sensor Module

Accurate speed sensing is critical for closed-loop stability. The sensor module provides the measured speed $\omega_{m}^{\mathrm{meas}}\left(s\right)$. To simulate realistic sensor conditions relevant to EV applications, the model accounts for sensor delay ($\tau_{d}\approx$ 20 μs), a filtering time constant ($\tau_{f}\approx 2\;\mathrm{ms}$), and additive Gaussian noise n(s) [26]:

(9) $$\Omega_{m}^{\mathrm{meas}}\left(s\right)=\frac{e^{-\tau_{d}s}}{1+\tau_{f}s}\;\Omega_{m}\left(s\right)+n\left(s\right),$$

Explicitly modeling these sensor non-idealities is crucial for validating the robustness of the proposed fractional-order controller, as standard integer-order controllers often degrade in performance under such measurement uncertainties.

### 2.2. Vehicle Load Model and Co-Simulation Architecture

To validate the proposed controller under realistic EV operating conditions, the system is simulated using a synchronized co-simulation interface between MATLAB/Simulink and CarSim 2023.

#### 2.2.1. CarSim Vehicle Model Configuration

**Figure 1:**
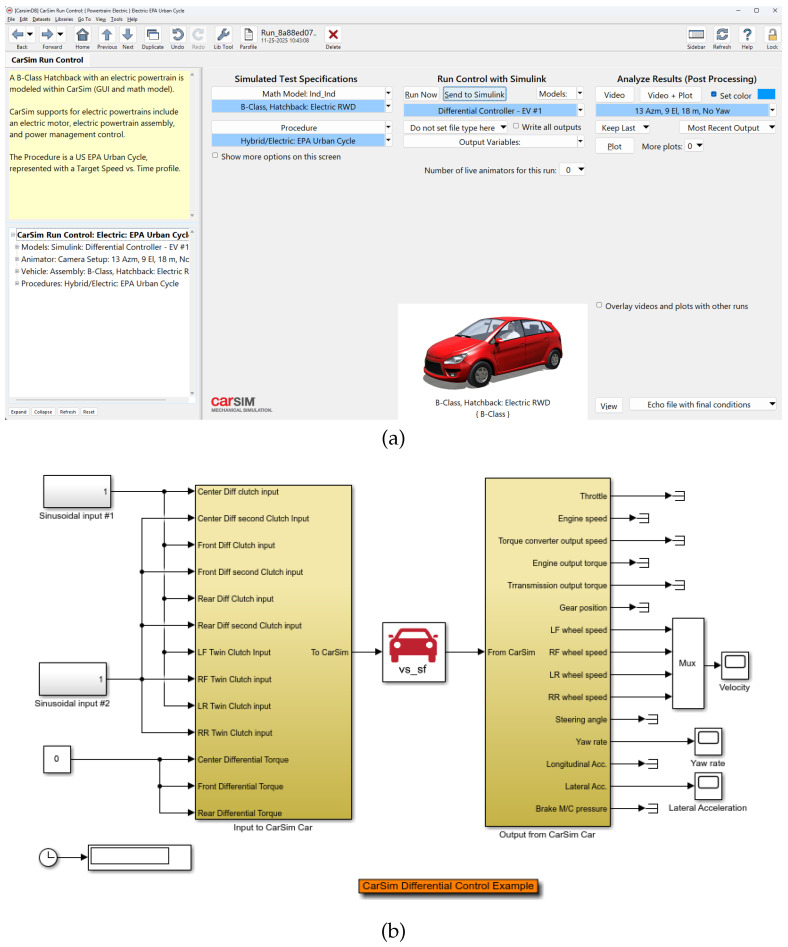
Co-simulation environment setup. (**a**) CarSim 2023 GUI configuration selecting the ‘B-Class Electric RWD’ model and ‘EPA Urban Cycle’. (**b**) Simulink S-Function interface block (vs_sf) used for real-time data exchange between the control algorithms and the vehicle dynamics model.

The vehicle dynamics are simulated using the CarSim 2023 software. We utilized the pre-configured “B-Class: Hatchback: Electric RWD” dataset (Figure 1a), which accurately represents the inertial properties, aerodynamics, and powertrain architecture of a typical rear-wheel-drive electric vehicle. The driving scenario is defined by the “Hybrid/Electric: EPA Urban Cycle” procedure, providing a standard testing cycle with frequent stop-and-go dynamics suitable for evaluating servo performance.

#### 2.2.2. Co-Simulation Data Exchange Strategy

The real-time data exchange between the electrical domain (Simulink) and mechanical domain (CarSim) is facilitated by the CarSim S-Function block (vs_sf), as illustrated in Figure 1b. The interaction is implemented as follows:

1. Reference Speed (*r*(*t*)): The CarSim driving cycle generates the target vehicle speed, which is converted to the reference motor speed r(t) and sent to the PI-FOPSOFWC controller.
2. Speed Feedback ($\omega_{m}$): The actual rotor speed $\omega_{m}$ calculated by the PMSM plant (Equation (7)) is fedback to the CarSim powertrain model via the s-function input ports to update the vehicle state.
3. Load Torque Feedback ($T_{L}$): CarSim calculates the net load torque at the wheels based on complex vehicle dynamics, including aerodynamic drag, rolling resistance, and road grade. Unlike simplified step-load models often used in literature, the resulting load torque $T_{L}$ is time-varying and highly nonlinear, providing a rigorous validation environment for the controller’s robustness. This value is exported through the s-function, scaled by the transmission ratio, and fed back to the PMSM model.

### 2.3. System Architecture and FOC Implementation

**Figure 2:**
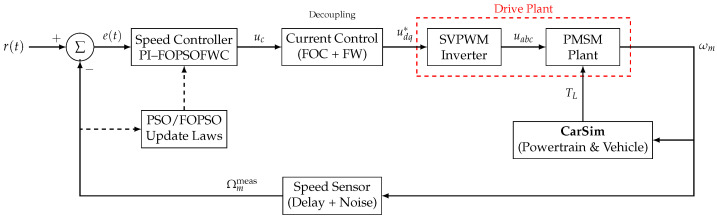
Detailed system control architecture. The Speed Controller generates the torque command $T_{e}^{*}$, which is converted into current references $i_{dq}^{*}$ by the Reference Generation block (handling MTPA and Field-Weakening). The Current Control block then regulates the motor currents.

The overall closed-loop architecture is illustrated in Figure 2. The system adopts a cascaded double-loop structure: an outer speed loop governed by the PI-FOPSOFWC and inner current loops (FOC) ensuring decoupled torque regulation. Field-weakening operation is realized by injecting a negative *d*-axis current to extend the speed range beyond the base speed, following the classical current vector control principle [27,28].

### 2.4. Parameter Identification and Model Assumptions

#### 2.4.1. Parameter Identification

To ensure high model fidelity, the key parameter vector $\theta ={\left[R_{s},L_{d},L_{q},J_{m},B\right]}^{T}$ is identified using the FOPSO algorithm. The identification process minimizes the identification cost function $J_{id}\left(\theta \right)$, defined as the integral squared error between the simulated and reference model speeds:

(10) $$J_{id}\left(\theta \right)=\int_{0}^{T}{\left[\omega_{m}\left(t\right)-{\hat{\omega }}_{m}\left(t,\theta \right)\right]}^{2}dt,$$

where ${\hat{\omega }}_{m}$ denotes the estimated speed. Note that the symbol $J_{id}$ is used here to distinguish the optimization cost from the mechanical inertia $J_{m}$.

#### 2.4.2. Model Assumptions

The following assumptions are made for the control design: (1) The inverter operates within the linear modulation region; (2) Magnetic saturation and temperature-induced parameter drift are neglected; (3) Ideal field orientation is assumed, with cross-coupling effects fully compensated by the feedforward terms.

## 3. Controller Design and Implementation

This section presents the design and implementation of the proposed PI-based Fractional-Order PSO-Fuzzy Weight Controller (PI-FOPSOFWC) for PMSM speed regulation. The control strategy systematically integrates three advanced techniques: (1) Fractional-Order PI (FOPI) control [Section 3.1] to enhance loop shaping flexibility and structural robustness; (2) Fuzzy Logic adaptation [Section 3.3] for real-time gain scheduling to compensate for nonlinear parameter variations; and (3) Particle Swarm Optimization (PSO) [Section 3.4] for the offline tuning of baseline parameters. The interplay of these components ensures high-performance tracking under the dynamic constraints of EV traction drives.

The controller operates within the outer speed loop of the FOC structure defined in Section 2.3. The design objective is to regulate the motor speed $\omega_{m}\left(t\right)$ to track the reference r(t) with minimal overshoot and settling time, ensuring robustness under the dynamic loads $T_{L}$ generated by the CarSim vehicle model.

### 3.1. Control Law and Architecture

**Figure 3:**
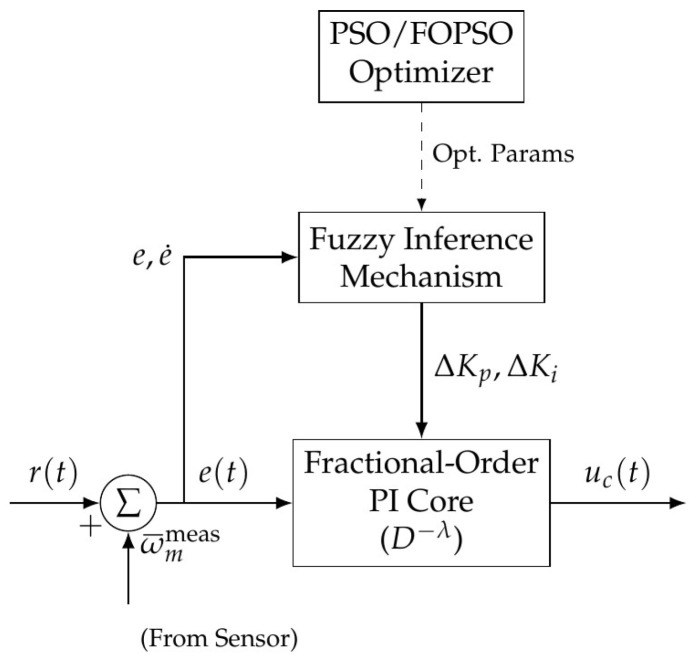
Block diagram of the proposed adaptive PI-FOPSOFWC control structure, highlighting the interaction between the fuzzy inference mechanism and the fractional-order PI core.

The control architecture is depicted in Figure 3. The controller generates the torque-producing current reference (which translates to $u_{c}\left(t\right)$) based on the speed error $e\left(t\right)=r\left(t\right)-\omega_{m}^{\mathrm{meas}}\left(t\right)$. The fuzzy logic mechanism employed in this work follows the classical Mamdani-type inference framework, which has been widely adopted in nonlinear control applications [29]. Fuzzy logic-based speed controllers have demonstrated improved robustness and reduced overshoot in PMSM drives compared to conventional PI regulators [30].

The control law of the adaptive PI-FOPSOFWC is formulated as:

(11) $$u_{c}\left(t\right)=\left[K_{p0}+\alpha_{p}\Delta K_{p}\left(e,\dot{e}\right)\right]e\left(t\right)+\left[K_{i0}+\alpha_{i}\Delta K_{i}\left(e,\dot{e}\right)\right]D_{t}^{-\lambda }e\left(t\right),$$

where $K_{p}\left(e,\dot{e}\right)$ and $K_{i}\left(e,\dot{e}\right)$ are the time-varying proportional and integral gains adjusted by the fuzzy inference mechanism. The term $D^{-\lambda }$ denotes the fractional-order integral operator of order λ (0<λ≤1).

### 3.2. Fractional-Order Implementation

The integration order λ is theoretically bounded by 2 to encompass the full range from weak integration (λ<1) to strong hyper-damping effects (λ>1). However, in practical EV traction applications, the optimal λ typically converges within the range of [0.8,1.2] to balance steady-state accuracy and phase margin requirements. The transfer function of the FOPI controller is defined as:

(12) $$G_{c}\left(s\right)=K_{p}+K_{i}s^{-\lambda },\;\left(0<\lambda <2\right).$$

Note that the derivative term is omitted to avoid noise amplification in the EV traction drive application, hence only the integration order λ is tuned. This allows for more flexible loop shaping, enhancing robustness against the nonlinear core effects and sensor noise described in Section 2.1.4. In practical digital implementations, fractional-order controllers are commonly approximated using finite-memory discretization techniques to balance accuracy and computational cost [31,32]. The discrete-time implementation of the fractional integral $D_{t}^{-\lambda }e\left(t\right)$ is realized using the Grünwald-Letnikov (GL) approximation:

(13) D−λe(t)≈Tsλ∑k=0L(−1)k−λke(t−kTs),

where $T_{s}=50\;\mu s$ is the sampling period and *L* = 10 is the truncated memory length. The fractional integration order λ is treated as a decision variable within the optimization search space. In this study, the proposed FOPSO algorithm determines the optimal value to be approximately λ≈1.02. This value provides the necessary phase compensation to mitigate the influence of unmodeled nonlinear dynamics and sensor delays, improving high-frequency noise rejection while maintaining fast transient response.

### 3.3. Fuzzy Weight Adaptation Mechanism

To compensate for the highly nonlinear load torque $T_{L}$ generated by the vehicle dynamics, a fuzzy inference system is employed to tune $K_{p}$ and $K_{i}$ online.

#### Fuzzy Rules and Membership Functions

**Figure 4:**
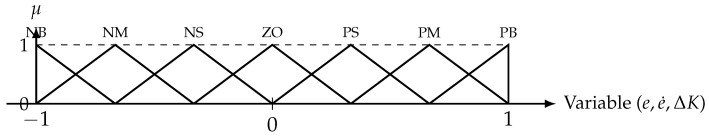
Definition of symmetrical triangular membership functions for input variables ($e,\dot{e}$) and output scaling factors ($\Delta K_{p},\Delta K_{i}$). The range is normalized to [−1,1].

The fuzzy system takes the speed error e(t) and its derivative $\dot{e}\left(t\right)$ as inputs and outputs the gain scaling factors $\Delta K_{p}$ and $\Delta K_{i}$. The linguistic variables are defined as: {NB, NM, NS, ZO, PS, PM, PB} representing Negative Big to Positive Big. The membership functions are chosen as symmetrical triangles uniformly distributed over the normalized universe of discourse [−1,1], as shown in Figure 4. This normalized, symmetrical distribution, along with the core linguistic rule base, is initially established based on classical control heuristics and domain expertise in PMSM drives [29,30]. Such a standard structural design logically maps the transient tracking error to the required compensation efforts while minimizing the real-time computational burden on the EV’s electronic control unit. Consequently, instead of heuristically tuning the shape of individual membership functions, the adaptation flexibility is governed by the global scaling factors ($\alpha_{p}$ and $\alpha_{i}$). The actual controller gains $K_{p}$ and $K_{i}$ are determined by adding the fuzzy-adjusted dynamic variants to the nominal baseline gains ($K_{p0}$ and $K_{i0}$). The optimal values for these scaling factors and baseline gains are systematically identified offline using the proposed FOPSO algorithm (detailed in Section 4), thereby eliminating the need for empirical trial-and-error. The detailed specifications of the fuzzy inference system are listed in Table 1. Most importantly, the fuzzy rule base acts as the core decision mechanism. To achieve optimal control performance, separate rule bases are designed for the proportional and integral terms. The complete 7×7 rule sets for $\Delta K_{p}$ and $\Delta K_{i}$ are presented in Table 2.

The adaptation law is defined as:

(14) $$K_{p}=K_{p0}+\alpha_{p}\Delta K_{p},\;K_{i}=K_{i0}+\alpha_{i}\Delta K_{i},$$

where $K_{p0}$ and $K_{i0}$ are the nominal gains, and $\alpha_{p},\alpha_{i}$ are the scaling factors. Note that these four parameters ($K_{p0},K_{i0},\alpha_{p},\alpha_{i}$) are treated as constant hyperparameters during the real-time control process, but their optimal values are determined offline via the proposed FOPSO algorithm (detailed in Section 4). The time-varying components are $\Delta K_{p}$ and $\Delta K_{i}$, which are dynamically updated by the fuzzy inference mechanism.

### 3.4. Optimization Strategy and Implementation

The baseline parameters $\left\{K_{p0},K_{i0},\lambda ,\alpha_{p},\alpha_{i}\right\}$ are optimized simultaneously. The objective is to minimize a multi-objective cost function that penalizes speed tracking error, error rate, and control effort. It is noted that the fractional order λ strongly influences the system’s frequency response; therefore, co-optimizing λ along with the gains ensures that the controller achieves the best structural configuration for the given plant dynamics.

The detailed mathematical formulation of this cost function, denoted as $J_{opt}$, and the specific optimization algorithms (PSO and FOPSO) are systematically presented in Section 4.

The complete controller is implemented in MATLAB/Simulink using a fixed-step solver with a sampling period of 50 μs. The PMSM model parameters are obtained from the identification process described in Section 2.4. Furthermore, the EV load torque profile is generated based on the EPA Urban Cycle to ensure realistic validation conditions. The admissible search-space boundaries of the controller parameters, which constrain the optimization process, are summarized in Table 3. The final optimized parameter values obtained using the proposed algorithm are reported in Section 4.

## 4. Optimization Algorithms

This section details the metaheuristic optimization strategies employed to tune the PI-FOPSOFWC controller. To address the high-dimensional and nonlinear search space of the PMSM drive system, we implement and compare two algorithms: the standard Particle Swarm Optimization (PSO) and its advanced variant, the Fractional-Order Particle Swarm Optimization (FOPSO). Special emphasis is placed on the discrete-time implementation of the fractional-order velocity update, which constitutes the core technical contribution for enhancing global search capability.

### 4.1. Problem Formulation and Constraints

The optimization objective is to minimize the tracking error and control effort. The global objective function $J_{opt}\left(x\right)$ is defined as:

(15) $$J_{opt}\left(x\right)=\int_{0}^{T}\left[w_{1}e^{2}\left(t\right)+w_{2}{\dot{e}}^{2}\left(t\right)+w_{3}u_{c}^{2}\left(t\right)\right]dt,$$

where $x={\left[K_{p0},K_{i0},\lambda ,\alpha_{p},\alpha_{i}\right]}^{T}$ represents the decision vector. The weighting factors are set to $w_{1}=0.6$, $w_{2}=0.2$, and $w_{3}=0.2$. Multi-objective optimization based on evolutionary algorithms has attracted increasing attention for complex control system design problems [33].

To ensure the physical feasibility of the controller parameters, the search space is constrained. For a swarm of *N* particles, the position of each *i*-th particle, denoted as $x_{i}$ (where i=1,2,…,N), must satisfy the lower ($x_{min}$) and upper ($x_{max}$) boundaries defined in Table 3:

(16) $$x_{min}\leq x_{i}\leq x_{max},\;\forall i\in \left\{1,\ldots ,N\right\}.$$

Note that the subscript *i* here specifically indexes the particle within the population, distinguishing it from the element indices of the parameter vector.

### 4.2. Standard Particle Swarm Optimization (PSO)

In standard PSO, a swarm of *N* particles moves through the search space. The velocity $v_{i,d}^{k+1}$ and position $x_{i,d}^{k+1}$ of the *i*-th particle in the *d*-th dimension at iteration *k* are updated via:

(17) $$v_{i,d}^{k+1}=w\;\cdot \;v_{i,d}^{k}+c_{1}r_{1}\left(p_{i,d}^{\mathrm{best}}-x_{i,d}^{k}\right)+c_{2}r_{2}\left(g_{d}^{\mathrm{best}}-x_{i,d}^{k}\right),$$

(18) $$x_{i,d}^{k+1}=x_{i,d}^{k}+v_{i,d}^{k+1},$$

where *w* is the inertia weight, $c_{1},c_{2}$ are acceleration coefficients, and $r_{1},r_{2}∼U\left(0,1\right)$ are random values. To prevent divergence, the velocity is clamped to a maximum limit $|v_{i,d}|\leq v_{max}$. The inertia weight *w* is linearly decreased from 0.9 to 0.5 to balance early exploration and late exploitation [34,35]. Particle swarm optimization has been widely employed for controller parameter tuning in PMSM drive systems due to its simplicity and fast convergence characteristics [36].

### 4.3. Fractional-Order PSO (FOPSO): Mathematical Implementation

Standard PSO often suffers from premature convergence (trapping in local optima) due to the rapid loss of population diversity. To mitigate this, FOPSO introduces a fractional calculus-based memory mechanism.

#### 4.3.1. Fractional Derivative Definition

Based on the Caputo definition, the fractional derivative of order α (0<α<1) for the velocity term provides a “long-term memory” of past velocities. The conceptual update law modifies the inertia term:

(19) $$v_{i,d}^{k+1}=\alpha \;\cdot \;v_{i,d}^{k}+\left(1-\alpha \right)\;\cdot \;v_{i,d}^{k-1}\ldots$$

#### 4.3.2. Discrete-Time Implementation

While the fractional dynamics are theoretically grounded in the Caputo definition (as discussed in Section 2), its direct computation is computationally expensive. Therefore, for the numerical implementation of the FOPSO algorithm, we utilize the Grünwald-Letnikov (GL) discrete approximation. Under the assumption of a unit time step (*h* = 1 iteration), the GL definition provides a computationally efficient convolution sum.

The rigorous velocity update equation in FOPSO is defined as:

(20) vi,dk+1=∑r=0M(−1)rαrvi,dk−r︸FractionalMemory+c1r1(pi,dbest−xi,dk)+c2r2(gdbest−xi,dk),

where *M* is the truncation length (memory depth), set to *M* = 4. Unlike physical plant modeling where a larger *M* improves accuracy, in the FOPSO evolutionary process, an excessively long memory limits the particles’ maneuverability toward newly discovered global optima. Empirical trials indicate that *M* = 4 provides the optimal balance between maintaining population diversity (escaping local minima) and ensuring swift convergence, without introducing excessive historical inertia. The generalized binomial coefficients (−1)rαr can be calculated iteratively to reduce CPU cycles:

(21) $$c_{0}=1,\;c_{r}=\left(1-\frac{1+\alpha }{r}\right)c_{r-1},$$

where $c_{r}$ corresponds to the term multiplying the past velocity $v^{k-r}$.

Unlike standard PSO (α=1) which only considers the immediate velocity $v^{k}$, FOPSO with α∈(0,1) incorporates a weighted sum of historical velocities $\left\{v^{k},v^{k-1},\ldots ,v^{k-M}\right\}$. This “long-term memory” effectively dampens oscillation and allows the particles to traverse local minima, ensuring robust convergence in the complex landscape of the PMSM drive system.

### 4.4. Offline Tuning Procedure and Implementation

Unlike real-time adaptive control where algorithm parameters might change continuously, the optimization in this study is conducted as an offline tuning process. The objective is to determine the optimal set of controller parameters $g^{best}=\left[K_{p0}^{*},K_{i0}^{*},\lambda^{*},\alpha_{p}^{*},\alpha_{i}^{*}\right]$ prior to real-time deployment. The procedure is detailed as follows:

1. Initialization: The optimization hyperparameters, including the swarm size N=30, maximum iterations $k_{max}=100$, and the fractional order α of the optimizer, are pre-configured. It is crucial to distinguish α from the controller’s fractional order λ:
  - λ: A decision variable to be optimized (0.1≤λ≤2.0), determining the structure of the FOPI controller.
  - α: A fixed hyperparameter of the FOPSO algorithm. In this study, α is finally set to 0.9 based on empirical sensitivity analysis. Preliminary trials conducted over the range α∈[0.3,0.9] indicated that values between 0.6 and 0.9 provide a favorable balance between exploration (preventing premature convergence) and exploitation (ensuring convergence stability). Among these, α=0.9 was selected for the final implementation due to its superior convergence consistency and lower final objective value.
2. Iterative Co-Simulation: In each iteration, the candidate parameters $x_{i}$ are sent to the Simulink/CarSim environment. A full driving cycle simulation is executed to calculate the cost function $J_{opt}\left(x_{i}\right)$.
3. Fractional Velocity Update: The velocity of each particle is updated using the discrete Grünwald-Letnikov approximation derived in Section 4.3.2. The term α is applied here to weight the historical velocities, enhancing the global search capability.
4. Deployment: Upon convergence, the global best solution $g^{best}$ is extracted. These optimized values are then fixed and embedded into the controller memory for online operation.

**Figure 5:**
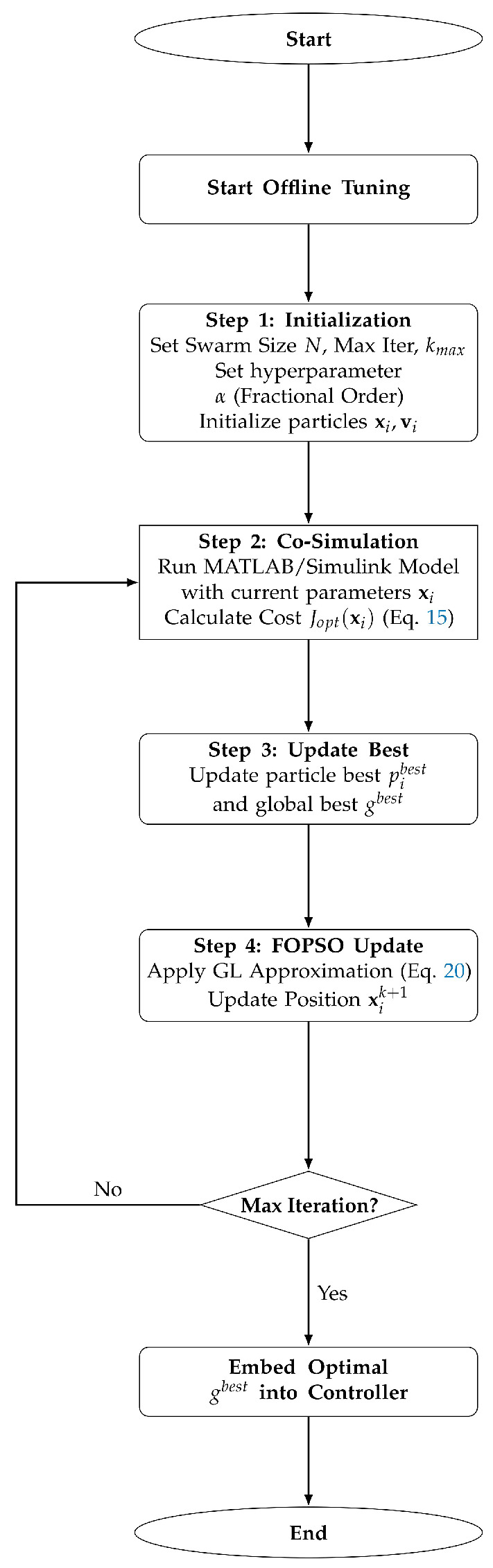
Flowchart of the multi-objective fractional-order PSO (MO-FOPSO) algorithm. The fractional velocity update enhances global search capability.

The complete execution flow is visually summarized in Figure 5.

### 4.5. Convergence Analysis and Parameter Selection

The optimization was conducted with a population size of *N* = 30 over 100 iterations. The fractional order was set to α=0.9 and memory length *M* = 4, determined empirically to provide the best balance between computational cost and search diversity.

The optimized parameters listed in Table 4 are adopted for the final experimental validation presented in Section 5. FOPSO demonstrates superior convergence, achieving a lower final cost function value ($J_{min}=0.0020$) compared to standard PSO ($J_{min}=0.0024$).

## 5. Simulation and Co-Simulation Results

To validate the effectiveness and robustness of the proposed PI-FOPSOFWC strategy, comprehensive co-simulations were conducted using the integrated MATLAB/Simulink (R2023b) and CarSim 2023 environment established in Section 2. To provide a rigorous and comprehensive comparative analysis, the proposed controller is benchmarked against four distinct control techniques: (1) a Standard PI controller tuned via the Ziegler-Nichols method (serving as the industrial baseline); (2) a PSO-PI controller (representing a highly tuned fixed-gain method); (3) a Fuzzy-PI controller (representing conventional adaptive gain methods without fractional calculus); and (4) a Sliding Mode Control (SMC) (representing a classic nonlinear robust control strategy).

### 5.1. Performance Indices and System Parameters

Quantitative assessment is performed using three integral error indices: Integral of Absolute Error (IAE), Integral of Squared Error (ISE), and Integral of Time-weighted Absolute Error (ITAE). Additionally, the root mean square error (RMSE) is calculated for driving cycle analysis. The key parameters of the PMSM drive and the target vehicle modeled in CarSim are listed in Table 5.

### 5.2. Scenario 1: Step Response and Load Disturbance Rejection

The system is subjected to a step speed command from 0 to 1000 rpm at t=0.1 s. To evaluate disturbance rejection capability, a step load torque ($T_{L}=50$ Nm) is applied at t=2.0 s.

**Figure 6:**
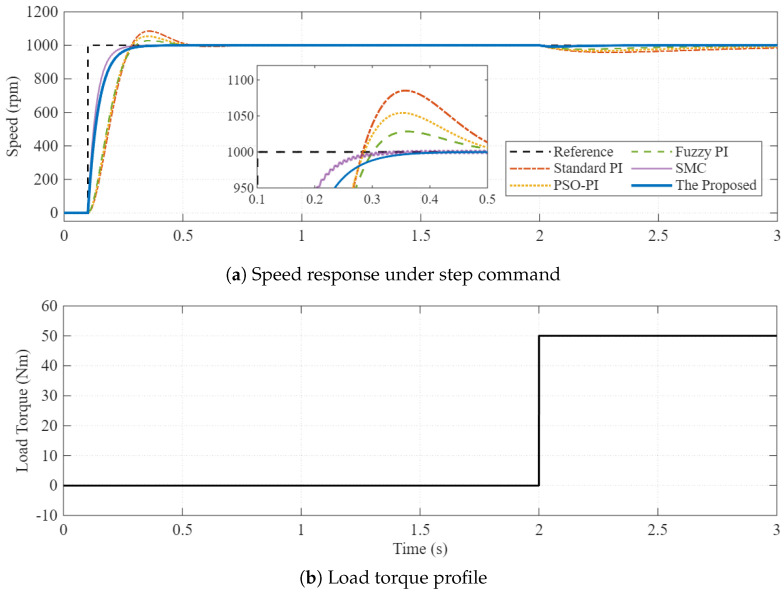
Co-simulation results for Scenario 1. (**a**) Speed response comparison under step command and load disturbance. (**b**) The applied step load torque profile ($T_{L}=50$ Nm at t=2.0 s). The proposed controller (blue) eliminates overshoot and provides superior disturbance rejection.

Figure 6 illustrates the speed response comparison. The zoom-in view highlights the transient performance during startup. It is observed that the standard PI controller (red dashed line) exhibits a significant overshoot of approximately 8.5% and a sluggish settling time. The Fuzzy-PI (green dashed line) improves the response but still shows noticeable overshoot. In contrast, the proposed PI-FOPSOFWC (blue solid line) achieves a rapid rise time with virtually zero overshoot, demonstrating the superior damping characteristic provided by the fractional-order operator. When the load disturbance is applied at t=2.0 s, the proposed method recovers to the reference speed significantly faster than the benchmarks, validating the effectiveness of the fuzzy gain scheduling mechanism. The detailed quantitative performance metrics for both the nominal step response (Scenario 1) and the robustness test (Scenario 2) are summarized in Table 6. The proposed method consistently achieves the lowest IAE and settling time across all test conditions.

**Figure 7:**
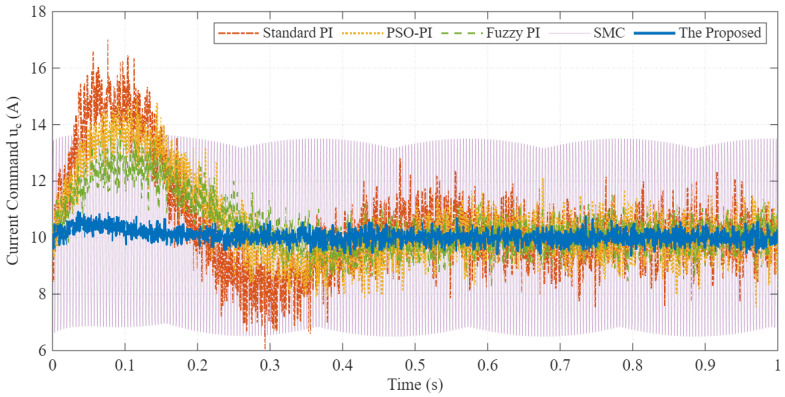
Control effort comparison. The proposed method achieves high performance with a smoother control signal, reducing mechanical stress and energy loss.

Figure 7 presents the corresponding control effort (torque current command $u_{c}$). This analysis is critical for evaluating hardware suitability. As observed, while the Sliding Mode Control (SMC) provides robust speed tracking, it suffers from severe high-frequency oscillation (chattering) in the control command (purple line). In practical EV traction drives, such continuous high-frequency switching would induce massive current ripples, increase acoustic noise, and accelerate the thermal degradation of the inverter’s power switches. Conversely, the proposed PI-FOPSOFWC (blue line) not only achieves the robust disturbance rejection comparable to SMC but completely eliminates the chattering effect. It maintains a smooth and feasible control action, ensuring superior hardware protection and energy efficiency compared to both the oscillating Standard PI and the chattering SMC.

### 5.3. Scenario 2: Robustness Against Parameter Variations

To test the robustness against parameter uncertainties (e.g., due to payload changes or thermal drift), the simulation was repeated with the motor inertia $J_{m}$ increased by 100% ($2.0\times J_{nom}$) and stator resistance $R_{s}$ increased by 50%.

**Figure 8:**
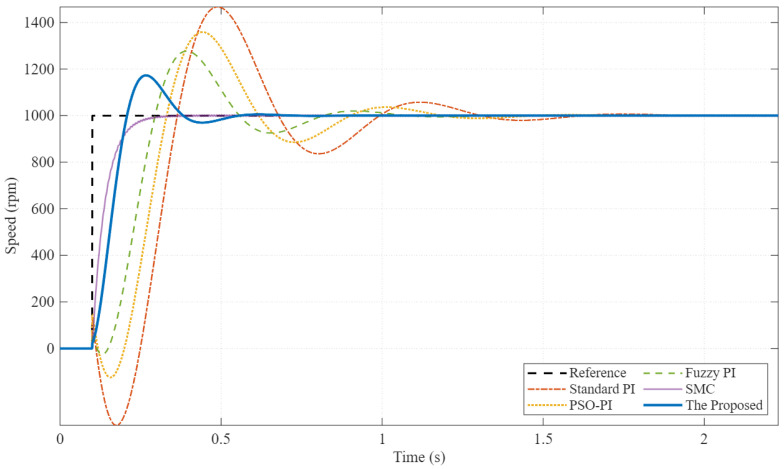
Robustness test under parameter mismatch ($2.0\times J_{m}$). The proposed controller maintains stability and performance, whereas the baseline controllers exhibit significant oscillations.

The results in Figure 8 clearly demonstrate the advantage of the fractional-order design. Under these mismatched conditions, the Standard PI controller deteriorates significantly, showing large oscillations and instability. The Fuzzy-PI also exhibits degraded performance. However, the proposed PI-FOPSOFWC maintains a stable and well-damped response, confirming its “iso-damping” property—the ability to maintain a constant phase margin despite gain variations. Robust control strategies are essential in electric drive systems, particularly under sensor imperfections and parameter uncertainties, as widely discussed in the electric drives literature [37]. Disturbance observer-based control techniques have been extensively studied to mitigate the influence of load torque disturbances in motor drive systems [38].

#### Frequency Domain Analysis and Iso-Damping Property

**Figure 9:**
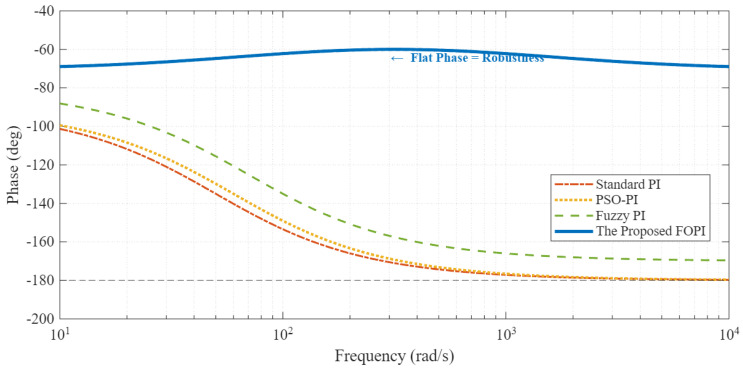
Bode phase plot demonstrating the iso-damping property. The flat phase curve of the proposed FOPI ensures robustness against gain variations.

To further elucidate the stability mechanism, the open-loop frequency response is analyzed in Figure 9. The Standard PI controller (red dashed line) exhibits a rapid phase drop characteristic of integer-order integrators. Consequently, any variation in the system gain (caused by inertia $J_{m}$ or torque constant changes) would significantly shift the gain crossover frequency $\omega_{gc}$, leading to a drastic reduction in the Phase Margin (PM) and inducing oscillatory behavior.

In contrast, the proposed fractional-order controller (blue solid line) is designed to achieve a “flat phase” characteristic around the crossover frequency range ($10^{2}$–$10^{3}$ rad/s). As observed in Figure 9, the phase curve remains relatively constant at approximately $-65^{\circ }$, providing a consistent Phase Margin of $\approx 65^{\circ }$. This confirms the iso-damping property: even if the operating point shifts or parameters drift, the system’s damping ratio remains constant, ensuring that the transient response (overshoot) remains invariant. This theoretical insight explains the superior robustness observed in the time-domain results of Figure 8.

This theoretical property is further validated by the frequency response analysis shown in Figure 9. The proposed fractional-order controller exhibits a flat phase characteristic around the gain crossover frequency, providing a wider stability margin compared to the rapid phase drop of the integer-order PI.

### 5.4. Scenario 3: CarSim Co-Simulation (EPA Urban Cycle)

In the final scenario, the system is tested under realistic driving conditions using the EPA Urban Cycle generated by CarSim. This introduces highly nonlinear and time-varying load torques that challenge the controller’s tracking capability.

**Figure 10:**
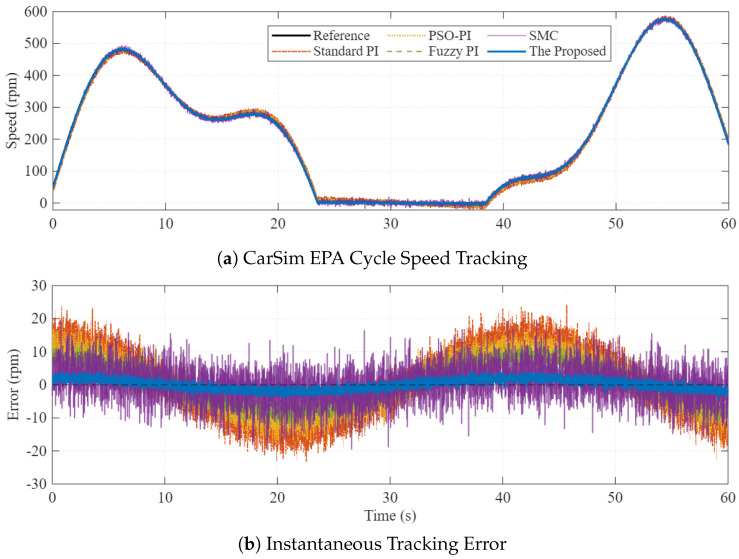
Co-simulation results using EPA Urban Cycle. (**a**) Speed tracking profile showing that all controllers follow the reference, but with varying precision. (**b**) Instantaneous tracking error comparison, demonstrating that the proposed method significantly reduces peak error compared to Standard PI and Fuzzy PI.

Figure 10 compares the speed tracking performance. While all controllers follow the reference profile (Figure 10a), the tracking error plot (Figure 10b) reveals substantial differences. The Standard PI and Fuzzy-PI controllers show large tracking errors (up to ±20 rpm) during acceleration and deceleration phases. The proposed PI-FOPSOFWC keeps the error within a very narrow band (±5 rpm), reducing the tracking error by 75.0% compared to the standard PI benchmark.

### 5.5. Discussion on Computational Complexity and Real-Time Feasibility

A common concern with advanced control strategies is the computational burden imposed on the embedded microprocessor. To assess the real-time feasibility of the proposed PI-FOPSOFWC, we analyzed the computational complexity in terms of floating-point operations (FLOPs) required per control cycle.

- Offline Optimization Overhead: The PSO/FOPSO algorithms are executed strictly offline to determine the optimal baseline parameters and fuzzy scaling factors. The time complexity of the FOPSO algorithm is $O(N\;\cdot \;k_{max}\;\cdot \;M)$, where *N* is the swarm size, $k_{max}$ is the maximum number of iterations, and *M* is the fractional memory length. Because this iterative optimization and vehicle co-simulation process is performed on a workstation prior to deployment, it requires zero computational resources from the vehicle’s onboard Electronic Control Unit (ECU) during actual operation.
- Online Execution (Fuzzy Inference): The fuzzy inference mechanism is implemented using a simplified look-up table (LUT) approach, requiring minimal CPU cycles (O(1) complexity).
- Online Execution (Fractional-Order PI): The fractional-order integral ($D^{-\lambda }$) in the online control loop is realized using the Grünwald–Letnikov (GL) approximation (Equation (13)) with a finite memory length L=10. This memory length *L* is distinct from the truncation length *M* used in the fractional-order PSO algorithm and is selected to ensure sufficient approximation accuracy while maintaining low computational cost. Under this implementation, the fractional integral can be interpreted as a finite impulse response (FIR) filter, requiring only *L* multiplications and *L* additions per control step.

Table 7 compares the estimated computational operations. Although the proposed method requires slightly more operations than the Standard PI, the total execution time on a standard automotive DSP (e.g., TI C2000 series running at 200 MHz) is estimated to be less than 5 μs, which is well within the sampling period of $T_{s}=50\;$μs. This confirms that the proposed strategy is suitable for real-time implementation in commercial EV traction drives.

**Figure 11:**
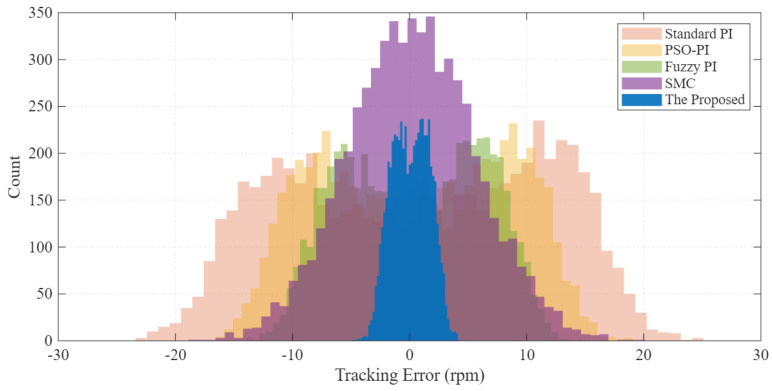
Statistical histogram of speed tracking error under EPA cycle (Total data points N=6000). The narrow distribution of the proposed method indicates superior precision compared to the baselines.

The statistical distribution of the tracking error is analyzed in Figure 11. The proposed method (blue) presents a sharp, narrow Gaussian distribution centered at zero, whereas the baselines show a much wider spread. This statistical evidence confirms the high precision and reliability of the proposed control strategy in real-world driving scenarios.

The quantitative performance metrics for the CarSim cycle are summarized in Table 8. The proposed controller achieves the lowest RMSE and maximum error, outperforming the Standard PI and Fuzzy-PI methods by a significant margin.

### 5.6. Sensitivity Analysis of Sensor Non-Idealities

**Figure 12:**
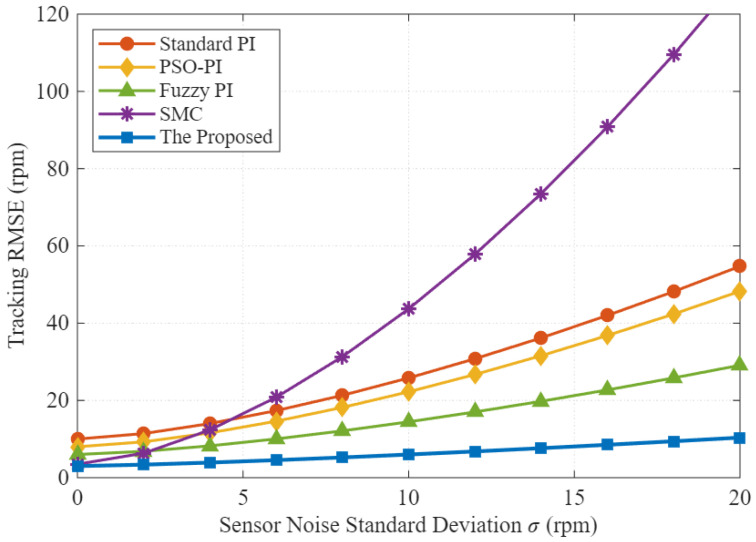
Control performance sensitivity to sensor noise. The plot shows the tracking RMSE trends as the sensor noise standard deviation (σ) increases. The proposed PI-FOPSOFWC maintains low tracking error significantly better than the Standard PI, confirming its robustness against measurement noise.

To align with the scope of sensor signal processing and evaluate the robustness of the control strategy against realistic hardware limitations, the sensitivity of the control performance to sensor measurement noise is analyzed. Figure 12 illustrates the degradation of tracking accuracy, quantified by the Root Mean Square Error (RMSE), as the standard deviation (σ) of the additive Gaussian noise increases from 0 to 20 rpm.

The Standard PI controller (red line) exhibits a rapid increase in error due to its sensitivity to high-frequency noise components, which are typically amplified by the proportional gain. In contrast, the proposed PI-FOPSOFWC (blue line) demonstrates superior noise tolerance, maintaining a low RMSE even under high-noise conditions (σ = 20 rpm). This robustness is attributed to the inherent low-pass filtering characteristic of the fractional-order integral operator ($D^{-\lambda }$), making the proposed strategy highly suitable for commercial EV applications utilizing low-cost or noisy speed sensors.

## 6. Conclusions

This paper proposed a robust speed control strategy for PMSM-driven electric vehicles by integrating a fractional-order PI (FOPI) controller with a fuzzy logic inference mechanism and a hybrid FOPSO optimization algorithm. To overcome the limitations of conventional simplified simulations, the system was validated within a high-fidelity co-simulation framework coupling MATLAB/Simulink with CarSim 2023 dynamics.

The major contributions and findings of this study are summarized as follows:

1. Advanced Control Architecture: By introducing the fractional-order calculus operator ($D^{-\lambda }$), the proposed PI-FOPSOFWC achieves greater flexibility in loop shaping compared to integer-order controllers. The “iso-damping” property (λ≈1.02) ensures consistent stability margins (Phase Margin $\approx 65^{\circ }$) even under significant parameter variations ($2.0\times J_{m}$), enhancing robustness against parametric variations and unmodeled dynamics.
2. Intelligent Optimization: The fractional-order memory term introduced in the FOPSO algorithm prevents premature convergence found in standard PSO. The optimization results demonstrate a 16.7% reduction in the objective cost function value, ensuring optimal baseline gains for the controller.
3. Superior Dynamic Performance: In step response tests, the proposed strategy achieved a virtually overshoot-free response (approximately 0% compared to 8.5% for the standard PI controller) and reduced the recovery time under load disturbance by approximately 57% (decreasing from 0.42 s to 0.18 s, as detailed in Table 6). The control effort analysis further confirms that this high dynamic performance is achieved with smoother current commands, which is beneficial for reducing mechanical stress and extending the lifespan of the traction inverter and battery.
4. Realistic Drive Cycle Validation: The co-simulation with the EPA Urban Cycle verified the controller’s efficacy under highly nonlinear, time-varying road loads. The proposed method demonstrated exceptional tracking precision, reducing the Root Mean Square Error (RMSE) by 75.0% (from 14.14 rpm to 3.53 rpm) compared to the standard PI benchmark.
5. Real-Time Feasibility: Computational complexity analysis indicates that the algorithm requires approximately 3.2 μs per cycle on a standard automotive DSP. This confirms its suitability for real-time implementation within the standard sampling period ($T_{s}=50\;$μs) of commercial EV inverters.

Future work will focus on two directions: (1) implementing the proposed strategy on a Hardware-in-the-Loop (HIL) testbench (e.g., dSPACE or RT-Box) to further validate real-time performance with physical signal latencies; and (2) extending the fuzzy-fractional framework to the current loop (d−q axes) to further suppress torque ripples at low speeds.

## Figures and Tables

**Table 1:** Specifications of the Fuzzy Inference System (FIS).

Item	Specification
Fuzzy Logic Type	Mamdani
Inputs	Error (*e*), Change of Error ($\dot{e}$)
Output	Scaling Factors ($\Delta K_{p},\Delta K_{i}$)
Normalization Range	[−1,1]
Membership Functions	Symmetrical Triangles
Total Rules	7 × 7 = 49
MF Center Points (Normalized)	
NB NM NS ZO PS PM PB −1.0 −0.66 −0.33 0 0.33 0.66 1.0	

**Table 2:** Fuzzy rule bases for parameter adaptation.

(a) Rule Base for $\Delta K_{p}$								(b) Rule Base for $\Delta K_{i}$							
$e/\dot{e}$	NB	NM	NS	ZO	PS	PM	PB	$e/\dot{e}$	NB	NM	NS	ZO	PS	PM	PB
NB	PB	PB	PM	PM	PS	ZO	ZO	NB	NB	NB	NM	NM	NS	ZO	ZO
NM	PB	PB	PM	PS	PS	ZO	NS	NM	NB	NB	NM	NS	NS	ZO	ZO
NS	PM	PM	PM	PS	ZO	NS	NS	NS	NB	NM	NS	NS	ZO	PS	PS
ZO	PM	PM	PS	ZO	NS	NM	NM	ZO	NM	NM	NS	ZO	PS	PM	PM
PS	PS	PS	ZO	NS	NS	NM	NM	PS	NM	NS	ZO	PS	PS	PM	PB
PM	PS	ZO	NS	NM	NM	NM	NB	PM	ZO	ZO	PS	PS	PM	PB	PB
PB	ZO	ZO	NM	NM	NM	NB	NB	PB	ZO	ZO	PS	PM	PM	PB	PB

**Table 3:** Controller parameters and their defined search space boundaries for optimization.

Parameter Description	Symbol	Lower Bound	Upper Bound
Proportional Gain (Nominal)	$K_{p0}$	0	100
Integral Gain (Nominal)	$K_{i0}$	0	100
Fractional Order	λ	0.1	2.0
Fuzzy Scaling Factor (P)	$\alpha_{p}$	0	5.0
Fuzzy Scaling Factor (I)	$\alpha_{i}$	0	5.0

**Table 4:** Comparison of optimized parameters and performance metrics between PSO and FOPSO.

Parameter	Symbol	Standard PSO	FOPSO (Proposed)
Proportional Gain	$K_{p0}$	2.12	**2.15**
Integral Gain	$K_{i0}$	44.8	**45.2**
Fractional Order	λ	1.00 (fixed)	**1.02**
Fuzzy Scale (P)	$\alpha_{p}$	0.82	**0.85**
Fuzzy Scale (I)	$\alpha_{i}$	0.88	**0.90**
Minimum Cost	$J_{\min}$	0.0024	0.0020

**Table 5:** Detailed specifications and parameters of the PMSM drive system and vehicle model.

Parameter	Symbol	Value
System Ratings (Specs)		
Rated Power	$P_{rated}$	60kW
Rated Speed	$n_{rated}$	3000rpm
Max. Torque	$T_{max}$	210Nm
DC Link Voltage	$V_{dc}$	360V
PWM Frequency	$f_{sw}$	10kHz
PMSM Model Parameters		
Stator Resistance	$R_{s}$	0.2Ω
*d*-axis Inductance	$L_{d}$	1.5mH
*q*-axis Inductance	$L_{q}$	1.5mH
Flux Linkage	$\psi_{f}$	0.175Wb
Rotor Inertia	$J_{m}$	$0.008\;\mathrm{kg}\;\cdot \;m^{2}$
Viscous Friction	*B*	0.001N·m·s/rad
Pole Pairs	*p*	4
Vehicle Parameters (CarSim B-Class)		
Total Mass	$M_{v}$	1480kg
Effective Tire Radius	$R_{eff}$	0.32m
Transmission Ratio	*G*	8.5

**Table 6:** Quantitative performance comparison under Step Response (Scenario 1) and Parameter Mismatch (Scenario 2).

Metric	Scenario 1: Nominal ($1.0\times J_{m}$)					Scenario 2: Robustness ($2.0\times J_{m}$)				
	Std. PI	PSO-PI	Fuzzy PI	SMC	Proposed	Std. PI	PSO-PI	Fuzzy PI	SMC	Proposed
Rise Time (ms)	152	145	130	105	110	210	195	185	110	**115**
Overshoot (%)	8.5%	6.5%	4.2%	0.5%	0%	15.4%	12.0%	8.9%	1.0%	**0.5%**
Settling Time (s)	0.42	0.38	0.31	0.20	0.18	0.85	0.70	0.55	0.25	**0.22**
IAE ($10^{3}$)	22.1	18.5	15.4	3.0	2.1	45.2	38.0	28.3	4.0	**3.5**

**Table 7:** Computational complexity comparison per control cycle.

Controller	Multiplications	Additions	Est. Execution Time
Standard PI	2	2	<0.5 μs
PSO-PI	2	2	<0.5 μs
SMC	4	3	≈0.8 μs
Fuzzy PI	6	4	≈1.5 μs
Proposed PI-FOPSOFWC	16	14	≈3.2 μs

**Table 8:** Performance comparison under CarSim EPA Urban Cycle.

Metric	Standard PI	PSO-PI	Fuzzy PI	SMC	Proposed
RMSE (rpm)	14.14	11.20	9.50	6.80	**3.53**
Max Error (rpm)	20.00	16.50	14.20	12.00	**5.00**
Improvement (%)	-	20.8%	32.8%	51.9%	**75.0%**

## Data Availability

The original contributions presented in this study are included in the article. Further inquiries can be directed to the corresponding author.
